# Risk factors associated with quadbike crashes: a systematic review

**DOI:** 10.1186/s13017-022-00430-2

**Published:** 2022-05-26

**Authors:** Preetha Menon, Marwan El-Deyarbi, Moien AB. Khan, Rami H. Al-Rifai, Michal Grivna, Linda Östlundh, Mohamed EI-Sadig

**Affiliations:** 1 Institute of Public Health, College of Medicine and Health Sciences, United Arab Emirates University, Al Ain, United Arab Emirates; 2 Department of Pharmacology, College of Medicine and Health Sciences, United Arab Emirates University, Al Ain, United Arab Emirates; 3 Department of Family Medicine, College of Medicine and Health Sciences, United Arab Emirates University, Al Ain, United Arab Emirates; 4 National Medical Library, College of Medicine and Health Sciences, United Arab Emirates University, Al Ain, United Arab Emirates

**Keywords:** Quadbike, All-terrain vehicle, Injury prevention, Risk, Safety, Haddon matrix, Sustainable development goals

## Abstract

**Background:**

Quadbikes or all-terrain vehicles are known for their propensity for crashes resulting in injury, disability, and death. The control of these needless losses resulting from quadbike crashes has become an essential contributor to sustainable development goals. Understanding the risk factors for such injuries is essential for developing preventive policies and strategies. The aim of this review was to identify the risk factors associated with quadbike crashes at multiple levels through a systematic review of a wide range of study designs.

**Methods:**

The study incorporated a mixed-method systematic review approach and followed the PRISMA 2020 guidelines for reporting systematic reviews, including a peer reviewed  protocol. This systematic review included observational studies investigating the risk factors associated with quadbike crashes, injuries, or deaths. Seven electronic databases were searched from inception to October 2021. Studies were screened and extracted by three researchers. Quality appraisal was conducted using the Mixed Methods Appraisal Tool (MMAT). Due to extensive heterogeneity, meta-analysis was not conducted. All the risk factors have been presented in a narrative synthesis for discussion following the guidelines for Synthesis without Meta-analysis (SWiM).

**Results:**

Thirty-nine studies combining an aggregate of 65,170 participants were included in this systematic review. The results indicate that modifiable risk factors, such as the increasing age of driving initiation, reducing substance use, and the use of organized riding parks, could reduce quadbike injuries. Riding practices such as avoiding passengers, avoiding nighttime riding, and using helmets could significantly reduce crashes and injuries among riders. Vehicle modifications such as increasing the wheelbase and limiting engine displacement could also help reduce crash incidence. Traditional interventional methods, such as legislation and training, had a weak influence on reducing quadbike injuries.

**Conclusion:**

Multiple risk factors are associated with quadbike injuries, with most of them modifiable. Strengthening policies and awareness to minimize risk factors would help in reducing accidents associated with quadbikes.

*PROSPERO registration number* CRD42020170245

## Background

Quadbikes or all-terrain vehicles (QB) are saddle-seated, four-wheel vehicles commonly used as off-road vehicles for farming and recreational purposes [1, 2]. However, QB are known for their increased instability while driving, most likely due to their high center of gravity, which lends the vehicle a higher propensity to tilt or roll over, leading to rider ejections and injuries, especially when driven off the road or in an uneven terrain [3]. To control these needless losses, several studies in numerous countries have attempted to identify the risk factors related to quadbike crashes and the resulting injuries and deaths [4–7]. These efforts have gained further momentum as the control of non-intentional injuries is increasingly considered one of the essential prerequisites to achieve the UN sustainable development goals (SDGs) by 2030 [8]. The UN’s SDG agenda has pushed many governments to prioritize and control injuries and occupational and recreational injuries using systems-level injury prevention strategies involving policy-makers, industry, and enforcement agencies [9–15].

It is therefore important to set up plans to observe and control QB injuries using well-designed prevention strategies and interventional programs [16]. However, a holistic understanding of the determinants and risk factors leading to quadbike crashes and the resulting injuries and deaths, both at the individual and at the system levels, should be achieved [17]. Risk mapping is a prerequisite for any such intervention design [17, 18]. To achieve that, public health scholars have attempted to identify risk factors through the analysis of injury databases, case–control studies, or case series. Unfortunately, these study designs are known to have limitations of temporality, small sample size, and inherent weaknesses in identifying and determining risk factors [19]. Since injury occurs due to a complex interaction of multiple risk factors, a systematic review of the literature is essential to identify those risk factors from different study designs and approaches.

Systematic reviews are employed to synthesize the known knowledge in the field, identify research gaps and priorities, and generate evidence for policy decisions [20]. However, they have seldom been used for risk identification or intervention design [21–23]. This systematic review attempts to develop a transparent and systematic method to identify the risk factors for quadbike crash injuries for future interventions.

Previous systematic reviews on QB injuries have either focused only on the pediatric population [5, 24] or have limited themselves to a few risk factors, such as helmet use [25] or a narrative literature review [26]. Unlike the narrative review that focuses on the rider risk factor, we have identified extrinsic and system-level risk factors that are not addressed in current interventions. This review incorporated a mixed-method systematic review approach [27] to capture the sources of extrinsic risk factors at the systems level.

The aim of the study was to identify the determinants and the intrinsic and extrinsic risk factors of QB crashes leading to injury and death together with determining the protective factors associated with those crashes. This systematic review was a risk identification exercise to compile evidence to design interventions aiming to control and reduce QB injuries.

## Methods

The study followed the Preferred Reporting Items for Systematic Reviews and Meta-Analyses (PRISMA) 2020 guidelines for reporting systematic reviews [20] and was prospectively registered online with Prospero [CRD42020170245]. A detailed peer-reviewed study protocol with a preliminary search strategy and inclusion and exclusion criteria has been published elsewhere [28].

### Eligibility criteria

We followed the protocol by including all observational study designs that studied the determinants and risk factors associated with crashes, injuries, or deaths among QB drivers of all regions, ages, and occupations. We also included studies with outcomes ranging from loss of control, injuries, or death. Editorials and reports citing primary research were excluded from extraction but screened for primary studies.

### Information sources

The search was initially conducted in June 2020 and was updated twice in March 2021 and on October 6, 2021, to include new publications (LÖ and PM). Five biomedical databases, including PubMed (the US National Library of Medicine), Embase (Elsevier), Scopus (Elsevier), APA PsycINFO (EBSCOhost), and Web of Science (Clarivate), were searched using the search string developed and reported elsewhere [28]. Additionally, the IEEE Xplore Digital Library (Institute of Electrical and Electronic Engineering) and ProQuest Dissertations and Theses (ProQuest) were also searched to cover relevant studies published in engineering and technolog y journals, dissertations, and theses. The systematic review software Covidence (Veritas Health Innovation, 2021) was used for automatic de-duplication, blinded screening, extraction of study characteristics and outcomes, export of data and references, and risk of bias assessment.

### Search strategy

LÖ and PM developed the search string from the Population–Exposure–Outcome research question, with two central functional units focusing on the target population and risk factors. The primary search string was systematically developed by using PubMed and PubMed’s MeSH. Search strings for other databases were fine-tuned using their thesaurus or subject headings and database-specific search rules. Pre-searches helped weed out keywords to improve the specificity of the search results. Gray sources like ProQuest Thesis and Dissertation were searched using shorter search strings when compared to electronic databases. The search strategy is detailed in the protocol paper [28].

### Selection process

After de-duplication, PM, MABK, and MED screened 1573 abstracts, while MS, MG, and RHA resolved conflicts during screening. One hundred twenty-one studies were reviewed for full text. Their PDF was uploaded into Covidence by the National Medical Library staff. However, we were unable to obtain full text for sixteen abstracts, nine of which were conference proceedings. PM and MABK selected 43 studies after blinded and independent full-text review, while MES resolved conflicts. We excluded two articles that shared the same data and results with other publications.

Moreover, we excluded studies that included (a) multiple recreational vehicles, (b) did not have an injury or crash outcome, or (c) did not test the association between risk factors and outcomes. These criteria eliminated many observational studies that reported risk factors without testing their association with outcomes. Adhering to the stringent inclusion criteria, we excluded research exploring riding behavior outcomes as they did not have injury or crash outcome data. Many studies relevant to intervention design were thus eliminated due to the systematic review protocol. Editorials and systematic reviews were excluded, but their references were scanned for relevant original research publications. References were hand-searched to identify potential articles that were missed by the search strategy.

### Data collection process

PM reviewed and extracted relevant data from 39 studies in Covidence software (Veritas Health Innovation, 2020). The extracted data tables were validated and checked by MED, MES, MG, RHA, and MABK. We ensured that two researchers reviewed each study. Furthermore, a mechanical engineering expert validated the evidence related to vehicle characteristics and simulation experiments. Finally, corrections were incorporated and validated again.

### Data items and effect measures

Study details and methods were documented along with risk data using Covidence software. The extracted data were limited to ris k factors, the outcome, and strength of association. When available, we recorded the confounders included in the model. Measures of association ranged from t-test and chi-square test to odds ratio, adjusted odds ratio, and risk ratio. Only those risk factors that had a significant association with the outcome were extracted and synthesized.

The studies selected for extraction had a wide range of outcomes. They included kinematic outcomes, such as loss of control, crashes with stationary objects, rollover, ejection, and collision, and injury-related outcomes such as hospitalization, head injury, general injury, musculoskeletal injury, head and neck injury, traumatic brain injury, and death. Since a wide range of study methods and study outcomes were involved in this review, we did not conduct a meta-analysis of the risk data.

### Risk of bias assessment

The risk of bias estimation and data extraction, utilizing the Mixed Methods Assessment Tool (MMAT) [29], was employed to assess the quality of the studies in terms of external validity, selection bias, measurement bias, and confounding. MMAT consists of a range of assessment tools for different study designs. PM, MED, and MABK critically appraised qualitative studies, quantitative non-randomized studies (retrospective analytical studies), and quantitative descriptive studies (survey) using different subscales of MMAT. The MMAT tool assesses each study with two standard probes on study objectives and four design-specific questions. Studies with an aggregated score of less than 50% were eliminated [27, 30–32]. Supplementary files show the MMAT scores of all the reviewed studies.

### Synthesis and Haddon matrix

We followed a narrative synthesis approach and the Synthesis without Meta-analysis (SWiM) reporting guidelines [27]. The extracted risk data were classified into intrinsic and extrinsic risk factors. Intrinsic risk factors were inherent, personal factors attributed to driver characteristics and riding behaviors. They formed the agent-based risk factors according to the Haddon matrix [33, 34]. The intrinsic factors (gender, age) were differentiated from the modifiable risk factors (e.g., helmet use). Modifiable risk behaviors are liable to change through interventions and hence were grouped separately [17, 35].

**Fig. 1:**
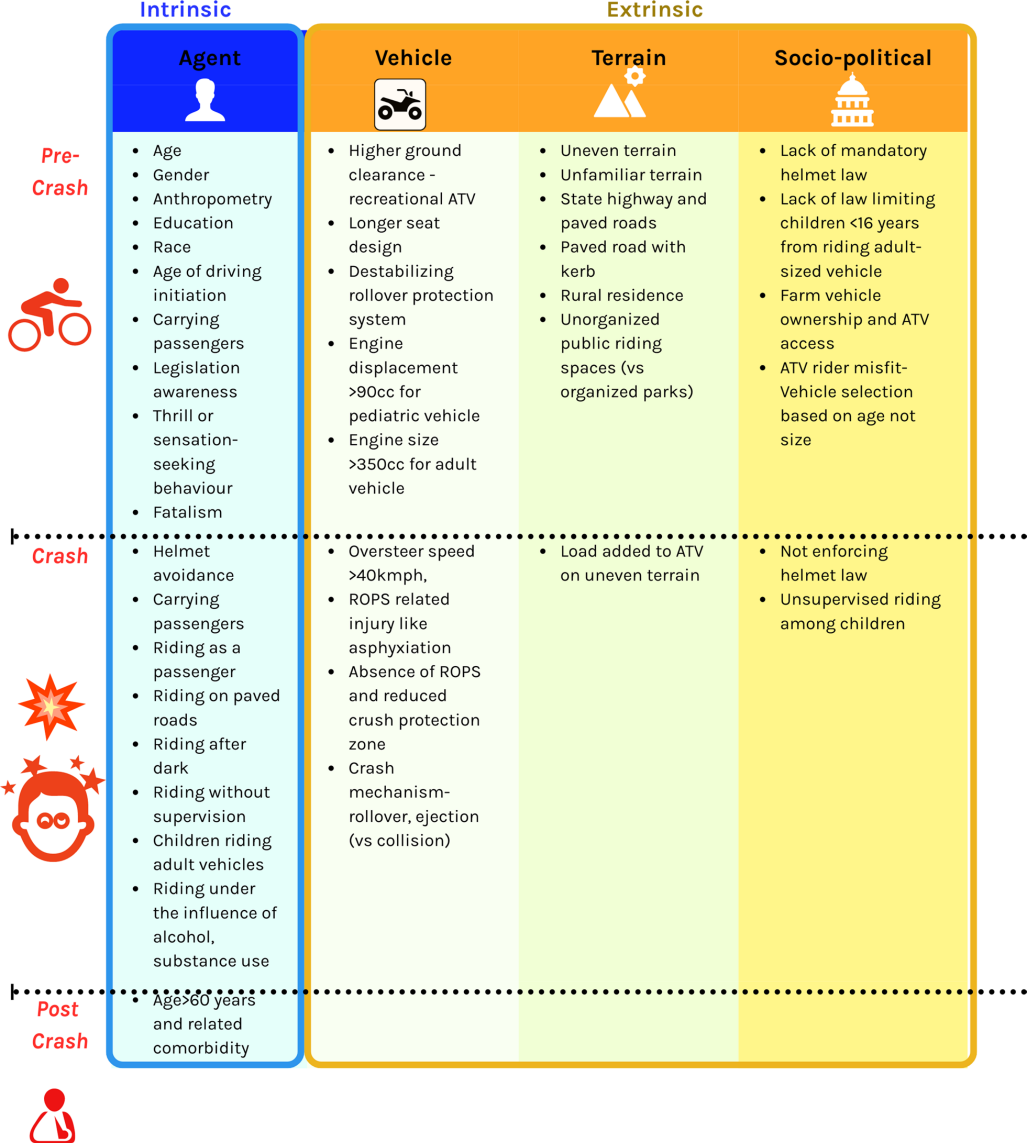
Haddon matrix: risk factors for crash, injury or death due to quadbike riding. Risk factors associated with quadbike crashes are organized in the Haddon matrix. Factors are classified into rider-dependent (agent) intrinsic factors and riding environment-dependent (vehicle design, terrain, regulatory environment) extrinsic factors

Extrinsic risk factors are factors that influence the driving environment and, subsequently, the risk for injury [36]. These factors were further classified into vehicle-related factors, driving terrain-related factors, and sociopolitical factors, as seen in the Haddon matrix framework. Finally, these risk factors were further classified as pre-crash, crash, and post-crash factors, consistent with the taxonomy of the Haddon matrix [33, 37, 38]. The risk factor analysis, using the Haddon matrix, gave a concise depiction of the risk identification exercise attempted in this review (Fig. 1).

## Results

### Study selection

Our search on PubMed brought in 801 records, Embase retrieved 646, Scopus 1176, APA PsycINFO 52, and the Web of Science brought in 561 records for screening. IEEE Xplore Digital Library brought in 79 records and ProQuest Dissertations and Theses brought 12.

### Study characteristics

The systematic review involved synthesizing data from thirty-eight published articles and one thesis. Of these sources, twenty-one were retrospective analyses of injury surveillance data following subjects for an aggregated period of 2809 months. Nine articles were cross-sectional observational studies. Seven studies were laboratory-based QB simulation studies with injury as their primary outcome, while one study was qualitative. The thirty-nine publications resulted in an aggregate sample of 65,170 study subjects. Eighty percent of the observational studies were surveys targeting a farming or rural community. Of all age-groups, 12 studies were focused on children under 18 years [12, 32, 39–48]. Most studies reviewed were from Canada [43, 49–51], New Zealand [9, 52–54], Australia [1], and Sweden [2], with the USA being the predominant contributor, with twenty-seven studies. The risk factors identified by the review were reported using the Haddon matrix framework.

### Risk of bias in the study

Some studies, based on retrospective surveillance data, scored low because the analysis did not adjust for confounding [10, 12, 39, 49, 55–57]. One cross-sectional study had a weak sampling strategy and did not represent the target population [58]. The excluded studies had additional weaknesses of low external validity [27, 30–32].

### Results of individual studies

The summary list of reviewed studies is categorized based on the type of study design. The study designs included cross-sectional survey studies and experimental, computer-based crash simulation models, evaluating outcomes from exposure to different risk factors. Table 1 summarizes 21 retrospective studies analyzing data from trauma registries and injury surveillance systems. Nine cross-sectional surveys and one qualitative study on QB drivers are shown in Table 2. The analysis shows inferences from primary data and surveys on specific demographic groups and locations. Finally, Table 3 highlights experimental studies that explore vehicle design-related factors that increase the risk of loss of control, rollover, or crash. These are not population-based studies, but they bring out the risk factors through computational modeling or crash tests. The summary tables highlight the risk factors known to have significant associations with outcomes.

**Table 1:** Summary of reviewed studies [retrospective analytical]

References	Study location—geogra phic region	Age range, if more specific	Sample size	Duration of records (months)	Data source	Study outcome of interest	Risk factor
**Bowman et al.** [63]	USA	All age-groups	11,589	48	National Trauma Data Bank-American College of Surgeons	Traumatic brain injury, neck injury, mortality, pediatric injury	Helmet non-use
**Benham et al.** [64]	Charlotte, North Carolina, USA	12–46 years	304	48	Trauma Registry, Carolinas Medical Centre	Traumatic brain injury	Helmet non-use
**Bethea et al.** [56]	South-West Virginia, USA	20–47 years	1857	23	Trauma Registry at Level 1 Trauma Centre	Injury	Substance abuse, helmet non-use
**Bohl** [60]	USA	> 21 Years	1884	12	National Electronic Injury Surveillance System	Hospitalization	Substance abuse, age
**Brandenburg et al.** [57]	Tulsa, USA	0–34 years	193	40	Trauma Emergency Centre records, St. Frances Hospital	Rollover	Uneven terrain, age
**Deladisma et al.** [61]	USA	All age-groups	6308	768	National Trauma Data Bank	Death	Age
**Denning et al.** [72]	Iowa, USA	All age-groups	813	95	Department of Transportation [Crash]; Department of Natural Resources [off-road crash public land] State trauma Registry	Crash	Organized riding environment
**Denning et al.** [83]	Iowa, USA	< 18 years	3240	180	U.S. Consumer Product Safety Commission database	Death	Gender, passenger status, age, multiple rider, helmet non-use
**Flaherty et al.** [66]	Massachusetts, USA	0–34 Years	4119	132	Massachusetts General Hospital Electronic record	Hospitalization	QB laws on pediatric minimum age; engine size
**Garay et al.** [41]	Pennsylvania, USA	< 17 years	1912	131	Pennsylvania Trauma Systems Foundation	Musculoskeletal injury	Passenger status
**Jennissen et al.** [55]	USA	All age-groups	537	12	State Trauma Registry	Backward rollover	Multiple rider, passenger status
**Jennissen et al.** [62]	Iowa, USA	All age-groups	3752	215	Iowa ORV injury surveillance database	Nighttime crashes	Age, alcohol use, helmet use
**Krauss et al.** [49]	Alberta, Canada	18–40 years	459	120	Alberta Trauma Registry	Death	Type of crash—rollover or ejection vs collision; head injuries; alcohol consumption
**Li et al.** [40]	USA	8–15 years	6826	288	National Electronic Injury Surveillance System (NEISS)	Injury	Age
**McBride et al.** [42]	North Carolina, USA	< 18 years	88	60	Wake Forest University Baptist Medical Centre Trauma Registry	Head neck injury	Passenger status, helmet non-use
**McLean et al.** [73]	Canada	< 16 years	5005	228	Canadian Hospitals Injury Reporting and Prevention Program (CHIRPP) surveillance	Head injury	Age, helmet non-use, minimum age for QB use legislation
**McLean et al.** [50]	Canada	> 16 years	5002	10	Canadian Hospitals Injury Reporting and Prevention Program (CHIRPP) surveillance	Injury	Age < 16 years, helmet non-use
**Pelletier et al.** [51]	Alberta, Canada	2–82 years	435	63	Alberta Trauma Registry (ATR)	Head injury, death	Helmet non-use
**Rodgers **[59]	USA	All age-groups	2401	108	U.S. Consumer Product Safety Commission database	Death	Age, occupation residence, gender
**Upperman et al.** [12]	USA	< 17 Years	1342	192	US Consumer Product Safety Commission database	Death	Legal and regulatory standards
**Winfield et al.** [10]	Florida, USA	All age-groups	377	36	National Trauma Registry of the American College of Surgeons (NTRACS), Hospital Database	Injury	Age, helmet non-use, driving on public roads

**Table 2:** Summary of reviewed studies [cross-sectional analytical studies]

References	Study location-geographic region	Study population—demographics	Age range	Sample size	Study outcome of interest	Risk factor
**Burgus et al.** [44]	Kentucky, Indiana, Iowa, Ohio, Wisconsin, Missouri, USA	Adolescent farmers	12–20 years	624	Injury	Gender, agricultural residence, age of riding initiation
**Butts et al.** [71]	Alabama, Florida, Mississippi, USA	Trauma center patients with QB injury	11–69 years	61	Traumatic brain injury	Engine size
**Campbell et al.** [32]	Connecticut, USA	Adolescent farmers	10–17 years	238	Injury	Unsupervised riding, engine size, riding after dark, participating in races
**Clay et al. **[9]	Otago and Southland, New Zealand	Farm workers	> 16 years	216	Loss of control events	Unrealistic optimism, impulsive sensation seekers, age, gender
**Clay et al.** [52]	South Otago, New Zealand	Farm workers	18–74 years	112	Loss of control events	High job demand, gender
**Clay et al.** *[53]	Otago and Southland, New Zealand	Farm workers	17–85 years	216	Loss of control events	Fatalism, risk taking, thrill seeking, time pressure, multitasking, fatigue, stress
**Jennissen et al.** [45]	Iowa State, USA	School students	11–16 years	4320	Crash	Gender, riding on paved road
**Jennissen et al.** [58]	Boone, Iowa; Decatur, Illinois, USA	Farm workers	> 17 years	426	Crash	Riding on unpaved road, riding frequency, age, gender
**Jones and Bleeker** [46]	Arkansas, USA	Student—agricultural education programs	< 19 years	378	Injury	Multiple riders, riding frequency
**Milosavljevic et al.** [54]	South Otago, New Zealand	Farmers and farm workers in Otago region	16–67 years	130	Loss of control event	Height taller than 1.81 m, driving a greater mean distance (> = 26.6 km)

These studies involve primary data collection through surveys, except for * which is a qualitative study

**Table 3:** Summary of reviewed studies [experimental studies]

References	Vehicle	Type of simulation	Outcome	Risk
**Bernard et al.** [47]	Kawasaki KFX90, Honda TRX500FM	Static test with tilt table and live human model	QB rider anthropometric fit	Age-based vehicle selection
**Edlund et al.** [2]	Honda TRX500FA Foreman, GOES 320	Static stability test with tilt table and live human model	Static stability from tilt and rollover	Heavier ROPS, heavier rider, lower tyre pressure, narrow track width
**Hicks et al.** [1]	Honda TRX 500, Yamaha YFM450, CF Moto CF500, Polaris Sportsman 450, Suzuki Kingquad 400 ASI, Kawasaki KVF300, Kymco MXU300, Honda TRX250	Computer simulation using finite element (FE) model of QB and seated rider	Rider displacement	Oversteer speed
**Khorsandi et al.** [26]	2018 Honda Recon ES, 2018 Honda Rancher Manual, 2018 Honda Rancher 4 × 4, 2018 Honda Foreman Rubicon 4 × 4, 2007 Honda Rancher, 2018 Yamaha Grizzly, 2018 Yamaha Kodiak 450, 2018 Yamaha Kodiak 700, 2013 Polaris Sportsman 500 H.O, 2018 Polaris Sportsman 570 EFI, 2018 Polaris Sportsman SP 850 H.O., 2017 Kawasaki Brute Force 300, 2018 Suzuki King-Quad 500 Axi 4 × 4	Models of crush protection devices	Crush protection zone	Vehicle height, rollover protection system
**Mattei et al.** [48]	Polaris Trailblazer 250, Honda FourTrax 250	Dynamic field test (J hook, brake, bump) with 5 riders	Rollover, longitudinal displacement and ejection, bounce and vertical displacement	Rider arm span, rider body weight, vehicle design
**Thorbole et al.** [67]		Computational model of QB using finite element (FE) software MADYMO	Crash—forward flip, lateral rollover	QB with passengers
**Zellner et al.** [65]	Honda TRX 350	Crash simulation with crash dummies	Simulated Injury	Rollover protection system, helmet use

### Results of synthesis

Table 4 presents the personal risk factors that predisposed QB riders to crashes, injury, or death. The data were from studies exploring associations between personal risk factors and crash or injury outcomes. The nature of the relationship between study outcomes and risk factors is included in the measure of association. The confounders included in the risk models are also documented in the table. Only the risk factors that showed significant association with outcomes were included in the table. These risk factors were further classified according to the age-group of riders in the study, with a separate section dedicated to youth riders.

**Table 4:** Intrinsic risk factors related to quadbike riding

Risk category	Risk factor	Study outcome	Association	Confounders accounted for	Study
**Personal risk factors (all ages) For Crash, Injury or Death due to QB use**					
**Inherent attribute**					
**Gender**	Being Male	Loss of control event	Adjusted incident rate ratio = 4.874 (2.655–8.947)	Age, unrealistic optimism, impulsive sensation seeking	Clay et al., 2014, New Zealand
	Being male	Crash	aOR = 2.23 (1.32–3.77)	Gender, riding frequency, riding with passengers, riding on road	Jennissen et al., 2017, USA
	Being male	Loss of control events	Adjusted IRR 4.00 (2.15, 7.44)	Job demand, workplace satisfaction, colleague support, QB experience, farm type	Clay et al., 2014, New Zealand
	Being male	Death	Relative risk = 1.050 (1.011–1.090)	Usage rate, rural, population under 25, college graduate, non-Hispanic white, farm workers	Rodgers, USA, 2008
**Age**	Age (Every 1-year increase)	Loss of control event	Adjusted incident rate ratio = 0.98 (0.969–0.991)	Gender, unrealistic optimism, impulsive sensation seeking	Clay et al., 2014, New Zealand
	Age (10-year increase)	Hospitalization	aOR = 1.35 (1.22–1.48)	Gender, substance abuse	Bohl, 2010, USA
	Age < 12 years vs 12–17 years	Rollover	RR = 1.96 (1.68–2.27)		Li et al., 2020, USA
	Age < 16 years	Head injury	OR = 1.45 (1.19–1.77)		McLean et al., 2014, Canada
	Age > 16 years	Nighttime crash	30% cases among adults vs 14% among youth. Chi-square test (*p* < 0.0001)		Jennissen et al., 2020, USA
	Age 16–20 years vs 31–65 years	Crash	aOR = 1.95 (1.09–3.51)	Gender, riding frequency, riding with passengers, riding on road	Jennissen et al., 2017, USA
	Age 21–30 years vs 31–65 years	Crash	aOR = 2.14 (1.12–4.11)	Gender, riding frequency, riding with passengers, riding on road	Jennissen et al., 2017, USa
	Age > 60 years	Death	OR = 6.96 (3.75–12.92)	Gender, systolic blood pressure, Glasgow Coma scale, respiratory rate	Deladisma et al., 2008, USA
	Age 12–17-year-old vs age < 12 years old	Injury	RR = 2.16 (1.67–2.80)		Li et al., 2020, USA
	Age 12–17-year-old vs age < 12 years old	Laceration and bleeding	RR = 1.43 (1.23–1.66)		Li et al., 2020, USA
	Age 12–17-year-old vs age < 12 years old	Ejection from QB vs rollover	RR = 1.29 (1.15–1.45)		Li et al., 2020, USA
**Physical attribute**	Height taller than 1.81 m	Loss of control events	OR = 1.08 (1.02–1.14; * p* = 0.008)	Height, weight, distance traveled, mean velocity, vibration	Milosavljevic et al., 2011, New Zealand
	Wingspan (with handlebar angle more than 60°)	Rollover	J hook test		Mattei et al., 2011, USA
**Socioeconomic attribute**	Non-Hispanic white	Death	RR = 1.013 (1.004–1.023)	Usage rate, rural, population under 25, college graduate, male, farm workers	Rodgers, 2008, USA
	College graduate	Death	RR = 0.969 (0.942–0.996)	Usage rate, rural, population under 25, race, male, farm workers	Rodgers, 2008, USA
**Modifiable attribute**					
**Helmet use**	Unhelmeted riders	Death	aOR = 6.577 (1.428–30.300)	Age, gender, blood alcohol level	Pelletier et al., 2012, Canada
	Unhelmeted riders	Death	OR = 2.58 (1.79–3.71), *p* < 0.001	Age, gender, clustering of facility, blood transfusion, hypotensive patients	Bowman et al., 2009, USA
	Unhelmeted riders	Traumatic brain injury	OR = 2.99 (2.30–3.89), * p* < 0.001	Age, gender, geographic region, hypotension, blood transfusion at hospital	Bowman et al., 2009, USA
	Unhelmeted riders	Traumatic brain injury	OR = 1.85 (1.45–2.37)		Bethea et al., 2014, USA
	Helmet use	Traumatic brain injury	OR = 0.36 (0.14–0.94)	Age, gender, injury severity score, helmet use, intoxication status	Benham, 2017, USA
	Unhelmeted riders	Major head injury AIS > = 3	aOR = 2.297 (1.033–5.109)	Age, gender, blood alcohol level	Pelletier et al., 2012, Canada
	Unhelmeted riders	Neck injury	OR = 3.53 (1.26–9.91), * p* = 0.017	Age, gender, geographic region, hypotension, blood transfusion at hospital	Bowman et al., 2009, USA
	Helmet use	Simulated neck injury	Injury risk/benefit percentage = 9% (6%,-21%); *p* < 0.001		Zellner et al., 2014, USA
**Substance abuse**	Alcohol consumption	Death	RR = 2.33 (1.52–0.56)		Krauss et al., 2010, Canada
	Alcohol use	Nighttime crashes	Chi-square *p* < 0.001 (44% alcohol consumption vs 13%)		Jennissen et al., 2020, USA
	Substance abuse	Hospitalization	aOR = 5.60 (3.46–9.09)	Gender, age	Bohl, 2010, USA
	Substance use [alcohol, narcotics, cannabis, benzodiazepines, amphetamine, cocaine, barbiturates, ecstasy]	Musculoskeletal injury	OR = 1.31 (1.03–1.67)		Bethea et al., 2014, USA
**Multiple riders**	Presence of passenger during rollover	Backward rollover	OR = 2.5 (1.1–5.7)		Jennissen et al., 2016, USA
	Presence of passenger during crash	Crash	OR = 5.3 (2.5–11.7)		Jennissen et al., 2016, USA
	Being a passenger during crash or rollover	Crash or rollover	OR = 3.6 (2.0–6.5)		Jennissen et al., 2016, USA
	Presence of passenger	Crash—forward flip, lateral rollover			Thorbole et al., 2012, USA
**Paved road**	Riding on paved road	Crash	aOR = 4.83 (1.23–18.93)	Gender, riding frequency, riding with passengers, riding on road	Jennissen et al., 2017, USA
**Personal Risk factors (children < 16) For Crash, Injury or Death due to QB Use**					
**Inherent attribute**					
**Gender**	Being a male	Crash	aOR = 1.61 (1.39–1.91)	Gender, age, riding frequency, rurality, riding with passenger	Jennissen et al., 2014, USA
	Being a male	Injury, general	OR = 1.62; 1.05–2.5		Burgus et al., 2009, USA
	Being a male (< 6 years)	Death	OR = 0.37 (0.21–0.65) for < 6 years	Helmet use, location, collision mechanism [QB-QB, QB-Veh, QB-other, non-collision], being a passenger	Denning et al., 2014, USA
	Being a male (6–11 years)	Death	OR = 0.54 (0.35–0.83)		Denning et al., 2014, USA
	Being a male (12–15 year)	Death	OR = 0.55 (0.37–0.81)		Denning et al., 2014, USA
**Modifiable risk factors**					
**Riding initiation**	Age of initiation < 12 years	Injury, general	OR = 4.08; 2.43–6.86		Burgus et al., 2009, USA
**Multiple rider**	Being a passenger (age 6–11 years)	Death	OR = 3.56 (2.36–5.39)	Gender, helmet use, location, collision mechanism	Denning et al., 2014, USA
	Being a passenger and age < 6 years	Death	OR = 21.1 (11.9–37.6)	Gender, helmet use, location, collision mechanism	Denning et al., 2014, USA
	Being a passenger	Head neck injury	OR = 8.3 (1.6–43.3)	Period [law enactment], helmet use, age, mechanism of injury, gender, race	McBride et al., 2011, USA
	Being a passenger	Musculoskeletal injury	OR = 0.69 (0.56–0.85)	Unadjusted	Garay et al., 2017, USA
	Multiple riders (driver)	Injury, general	OR = 2.74 (1.13–6.65)	Age, race, QB ownership, training, frequency of operation, helmet use	Jones et al., 2005, USA
**Riding habit**	Riding on paved road	Crash	aOR = 1.77 (1.14–2.74)	Gender, age, riding frequency, rurality, riding with passenger	Jennissen et al., 2014, USA
	Riding after dark	Injury, general	Chi-square (*p* < 0.001)		Campbell et al., 2010, USA
	Frequency of QB use > 3 per week	Injury, general	aOR = 3.46 (1.48–8.08)	Age, race, QB ownership, training, frequency of operation, helmet use	Jones et al., 2005, USA
**Helmet use**	Unhelmeted riders	Head injury	OR = 1.6 (1.43–1.81)		McLean et al., 2014, Canada
	Unhelmeted riders (6–11 years)	Death	OR = 1.45 (1.04–2.02)	Gender, location, collision mechanism, passenger status	Denning et al., 2014, USA
	Unhelmeted riders	Traumatic brain injury	OR = 2.32 (1.23–4.37)	Age, gender, clustering of facility, blood transfusion, hypotensive patients. helmeted riders as reference	Bowman et al., 2009, USA
**Parental negligence**	Children riding without supervision	Injury	Chi-square (*p* < 0.001)		Campbell et al., 2010, USA
	Parents allowing children to ride adult QB	Injury	Chi-square (*p* < 0.001)		Campbell et al., 2010, USA

Table 5 synthesizes and presents the extrinsic risk factors that contribute significantly to an unsafe driving environment. The first category of vehicle-related factors is “design-related factors” that contribute to loss of control, rollover, collision, and ejection. The next category of “legislation” compiled results from studies exploring the influence of national- or state-level legislations aiming to enhance safe driving. Finally, the literature also pinpointed other “terrain-related risk factors” related to physical environmental features that may contribute to increasing crashes or injuries. The measures of risk association used in the study (e.g., relative risk, rate ratio, odds ratio, etc.) were also described in the comments section. All the results of the synthesis are displayed in the form of a Haddon matrix (Fig. 1) organized as pre-crash and crash factors.

**Table 5:** Extrinsic risk factors related to quadbike riding

Risk category	Risk factor	Study outcome	Comment	Study
**EXTRINSIC RISK FACTORS For Crash, Injury or Death due to Quadbike use**				
**Vehicle design**	Vehicle design—low ground clearance [utility vehicle Honda vs sport vehicle Polaris]	Rollover	Sports vehicle Polaris has a greater distance from its foot-peg to the seat, keeping the rider's legs more extended during sitting position. This gives less space to bounce or vertical buffering during a bump, increasing the risk of injury	Mattei et al., 2011, USA
	Vehicle track—width	Static tilt angle for lateral rollover	An increase in track width by 20 mm resulted in a stability of more than 32Â°	Edlund et al., 2020, Sweden
	Seat design accommodating for passenger	Forward flip, lateral rollover	QB with two riders is more unstable and more likely to roll in both flip forward and lateral rollover accidents	Thorbole et al., 2012, USA
	Rollover protection system	Crush protection zone during three types of rollover	Installation of Quadbar, Lifeguard, and Air-Quad systems increases the crush protection zone in case of a rollover, thus reducing the risk of injury to the trapped rider	Khorsandi et al., 2019, USA
	Rollover protection system—Quadbar use	Simulated injury, asphyxiation	Risk/benefit percentage for injury in unhelmeted rider = 492%[95% CI 255%, 788%]; *p* < 0.001	Zellner et al., 2014, USA
	Engine size > = 350 cc, when compared to < 350 cc	Outcome injury severity score	Injury outcome score among those riding with engine size > = 350 cc was 6.4 (*p* < 0.05) higher than those riding with engine size < 350 cc	Butts et al., 2015, USA
	Age, not height used as a determinant for QB size selection	Pediatric rider QB misfit	Older children [12–15 years] fitting adult sized QB better than youth sized QB. Young drivers (12–15 years) not meeting size parameters of youth-sized QB [taller children], 6–11 year old not meeting size parameters of adult QB	Bernard et al., 2010, USA
	Pediatric QB with engine size greater than 90 cc	Injury	Pediatric QB users were more likely to have experienced a crash when engine displacement is more than 90 cc (*p* < 0.01)	Campbell et al., 2010, USA
	Oversteer speed > 40kmph	Rider displacement and rollover	A 100-mm hump on paved roads can displace the rider from seated position when turning at a high speed	Hicks et al., 2017, Australia
**Legislation and implementation**	Minimum age limit 16 years for driving QB—Canada	Hospitalization	Decreased hospitalization rate after introduction of legislation, but not supported statistically	McLean et al., 2014, Canada
	Non-enforcement, violating state laws (Florida, USA)	Mortality rate	Significant difference in mortality rate (*p* = 0.045) between violators and non-violators of state laws of minimum age of 16 years, use of helmet and not driving on public roads	Winfield et al., 2010, USA
	States with QB safety certification and licensing laws	Pediatric mortality rate	There is no significant difference between high mortality states and other states with regard to safety certification, licensing laws. *p* < .61 and *p* < 0.07	Upperman et al., 2003, USA
	2010 Massachusetts ORV law for children	Hospitalization	ORV law (banning QB use for those under 10 years, limited use by 10–13 to events under parent supervision and engine size less than 90 cc) saw 41% drop (*p* < 0.001) in rates of inpatient hospitalization for 0–17 year age-groups after its implementation in 2010	Flaherty et al., 2017, USA
	2010 Massachusetts ORV law for children	Emergency department visit	33%, 50%, 39% decline in emergency department visits in 0–9 years; 10–13 years; 14–17 years age-group with *p* < 0.001. There was a net 28.5% drop in emergency department visit after the law implemented in 2010 with *p* < 0.001	Flaherty et al., 2017, USA
**Environment and terrain**	Uneven terrain	Rollover	A retrospective analysis showed greater risk of injuries when driving on uneven terrain with odds ratio = 32.9 (6.6–221.5)	Brandenburg et al., 2007, USA
	Unfamiliar terrain	Injury	This qualitative study highlights farmer perception of greater risk of injury when they travel on unfamiliar terrain	Clay et al., 2015, New Zealand
	Type of crash—rollover	Death	Retrospective analysis of severe trauma due to QB showed greater risk of death due to rollover when compared to collision RR = 2.75 (1.13–6.70)	Krauss et al., 2010, Canada
	Type of crash—ejection	Death	Retrospective analysis of severe trauma due to QB showed greater risk of death due to ejection when compared to collision RR = 4.28 (1.7–10.32)	Krauss et al., 2010, Canada
	Rural residence	Death	Riders residing in rural areas were at greater risk of death, when compared to urban residents with RR = 1.019 (1.007–1.031)	Rodgers, 2008, USA
	Farm vehicle ownership	Injury	A survey showed youth living in a farm had greater risk of injury if they owned a vehicle when compared to those who did not, with OR = 4.04 (2.08–7.86)	Burgus et al., 2009, USA
	Vehicle driven in public spaces vs organized riding parks	Crash (pediatric)	Children had lower risk of crash when driving in organized recreational parks than on public spaces (*p* < 0.01, chi-square test)	Denning et al., 2013, USA
	Vehicle driven in public spaces vs organized riding parks	Head injury GCS < 15	Children had greater risk of head injury when riding in public spaces than when compared to organized recreational parks (*p* < 0.0001, Fisher exact probability)	Denning et al., 2013, USA
	QB crash occurring in recreational parks	Death	Records of severe QB trauma showed higher risk of mortality when QB was driven in recreational parks when compared to home or occupational settings with RR = 3.66 [IQR, 2.52–5.32]; *p* < 0.000	Krauss et al., 2010, Canada
	QB crash occurring in state highways and paved surfaces	Death	Records of severe QB trauma showed higher risk of mortality when QB was driven on state highways and paved surfaces when compared to home or occupational settings with RR = 2.56 [IQR, 1.73–3.80]; *p* < 0.000	Krauss et al., 2010, Canada

OR = odds ratio; RR = relative risk

### Risk factors associated with quadbike crashes, injuries, and deaths

#### Intrinsic personal risk factors

##### Gender

The results show that males are more prone to QB crashes due to loss of control of the vehicle when compared to females of the same age or impulsive nature [9, 58]. Men were also at greater risk of dying due to QB crashes when compared to women, irrespective of age, education, race, or rural residence [59]. Rodgers also proved that this mortality rate was not due to frequent usage of quadbikes by men or boys after adjusting the model for usage rates [59]. Clay disproved the argument that being more impulsive and thrill-seeker makes male riders more prone to crashes. He found a fourfold increase in the risk for loss of vehicle control among males despite being adjusted for behavioral factors such as thrill-seeking and impulsiveness [9]. In contrast, Denning observed that girls, less than 15 years old, were at a higher risk of death than boys [39].

##### Age

Younger drivers were at a higher risk for loss of vehicle control, which increased by 20% for every 10-year decrease in age [9]. However, this increased risk for crashes was not accompanied by a similar risk for hospitalization, compared to older riders who had an increased risk of hospitalization with increasing age [60]. Similarly, the risk of death was much higher among riders older than 60 years [61]. The risk among children shows variation among different age-groups. Older children (12–17 years) showed a greater risk for injury, crash, and ejection than younger children (< 12 years) [40]. In contrast, riders aged 16 years and above were more prone to nighttime crashes than younger riders [62]. Different studies categorized children into different age-groups, with 16 years being the legal age for transitioning to adult-sized bikes.

##### Age of riding initiation and riding transition

The data showed that young riders are introduced to the QB as passengers before they become riders themselves. However, children who start riding QBs at ages less than 12 years are four times more likely to suffer from QB injuries than those who start riding at an older age [44]. Contrary to the notion that younger riders were more at risk for crashes due to their smaller stature, Milosavljevic observed that riders taller than 1.8 m were at a higher risk of losing control of vehicles [54]. The physical attributes of the rider and rider-vehicle fit were explored in numerous studies [1, 4, 59, 68, 69] and are currently considered prerequisites for active riding. Active riding involves continuous movement of arms, legs, and torso to keep the vehicle in control. The rider must actively change his body position to avoid rollover.

The QB design for active riding comes with longer seats, which reduces the distance from the tip of the seat to the handlebars. These design features compromise grip strengthening for braking or control or arm span for sharp turns when small children operate adult-sized vehicles with higher engine displacement. The longer seat design encourages pediatric use and predisposes them to loss of control and crash [47, 58]. Campbell observed that children under the age of 16 driving adult-sized vehicles were more likely to experience crashes [32]. This observation was contested by Bernard who observed that anthropomorphic fit is a better parameter to decide vehicle transition than age. He observed that taller and older children were at a higher risk of crashes on a child-sized QB than an adult one, thus challenging the age-based criteria for riders to transition to adult QBs [47].

##### Helmet use

Several studies have shown that helmet nonuse among riders predisposes them to more severe head injuries, traumatic brain injuries, and death [51, 56, 63–65]. Helmets are known to reduce the severity of head and neck injuries and crash injuries occurring during rollover [66]. This protective effect was more observable among children, where unhelmeted children had a five times higher risk of severe head and neck injury [42].

##### Multiple riders

Multiple riders pose an additional risk for QB safety. Multiple riders occur when an active rider holding on to the handlebars takes on one or more passengers. These passengers are not as involved as the rider in controlling the vehicle. A passenger crash impact kinematic study reported that additional passengers make QBs more unstable and more predisposed to rollovers and forward flips [67]. Though intended for a single rider, the quadbike seats are designed long enough to enable active riding. Nevertheless, riders misuse this feature and keep taking on passengers. In fact, taking passengers adversely affects active riding mobility, especially when driving up or down a slope, making them more at risk of rollover and crash [67]. A retrospective analysis of injury data showed higher odds of backward rollovers and crashes among riders who had taken in multiple passengers [46, 55]. In contrast, taking on passengers also showed a protective effect with multiple riders preventing driver ejection in the event of a collision [55]. This observation is supported by static stability tests that showed compromised vehicle stability with multiple passengers or greater passenger weight and slower shift in the center of gravity [2].

Being a passenger was also a risk factor for injury, with passengers reported to have higher odds of experiencing injury during crashes or rollovers than drivers. Jennissen also observed that children under 15 and females were more likely to ride as passengers [42]. Furthermore, the risk of death increased from 3.56 to 21 when the passenger age was less than six years compared to those aged 6–11 years [39]. This risk for severe injury is compounded because multiple riders are less likely to be helmeted compared to single riders [55].

##### Substance abuse

Driving under the influence of drugs or alcohol is known to be significantly associated with reckless, speedy driving, crashes, and fatal injuries. Substance abuse impairs cognition, perception, attention, balance, coordination, and other brain functions that are necessary for safe quadbike driving. The active riding of quadbikes involves a constant positional adjustment in reaction to shifting terrain [1], which could be compromised if the driver is intoxicated. Moreover, intoxicated drivers are less likely to brace for a crash or rollover. These conclusions were drawn by Benham who noted that intoxicated riders are more likely to sustain severe injuries to the thorax, spine, and brain. He also noted that non-intoxicated riders were more likely to have less severe injuries [64]. Other studies showed similar observations of a higher risk for musculoskeletal injury, hospitalizations, and death among riders under the influence of alcohol, narcotics, etc., especially when riding at night [49, 56, 60, 62]. Intoxicated riders were also four times less likely to be helmeted [62, 64], increasing the risk for severe injuries.

##### Driving speed

Speed is known to be a major risk for road traffic crashes [68]. Additionally, it is known that the higher the driving speed, the higher the collision speed that leads to severe injuries. This is because riders have less reaction time for protective action and, therefore, lower likelihood of avoiding crashes. The same applies to driving quadbikes, especially on uneven terrains. For example, an observational study, by Hicks et al., reported a greater likelihood of rider displacement on uneven surfaces when the oversteer speed was more than 40 km per hour (kmph) [69]. When driving at 20 kmph on a slope of 12°, even an obstacle of 100 mm (approximately the length of the long edge of a credit card) can tip a vehicle over [2], showing the instability inherent in recreational QBs. Farmers have observed high speed as a risk factor when maneuvering a vehicle with sudden brakes or sharp turns [53].

##### Distraction and multitasking

Long working hours, stress and time pressures, multitasking, and fatigue are all known risk factors, causing farmers to make poor driving choices and judgments leading to quadbike crashes. This risk pathway is specific to farmers but different than that witnessed with thrill seekers, as farmers are presumably aware of the additional risk associated with their driving choices [53].

##### Impulsive and thrill-seeker drivers

A study among farmers revealed that younger male riders with impulsive and thrill-seeking tendencies were more likely to experience a loss of control of the vehicle leading to crashes [9]. Paradoxically, however, loss of control events were higher prevalent among riders who perceived a higher susceptibility to crash [53]. This is contrary to the health belief model where the perception of higher risk leads to the adoption of safe riding behavior and subsequently a lower susceptibility to crashes.

##### Experience and training

Rider inexperience in handling quadbikes and active riding techniques are known to predispose farmers to lose control of their vehicles [53]. In contrast, education and training did not reduce the loss of control events, an observation attributed to higher reporting among those trained [9]. Similarly, Jones found that QB safety education and training are ineffective in reducing injuries among young QB riders [46], adding to the ambiguity of focusing on education and training as effective interventional safety measures.

#### Extrinsic factors

##### All-terrain vehicle-related factors

Injury during quadbike riding occurs when the rider loses control, resulting in the vehicle rollover or colliding with another object. Rollovers or collisions are known to throw riders off the vehicle, which is also known as ejections [55]. Active riding on uneven surfaces at higher speeds requires constant movement of arms, legs, and torso, making seatbelts redundant. A lack of seatbelts or rider protection equipment makes the rider more prone to ejection during a collision or a crash [67]. It also carries the greatest risk of trapping the rider during a rollover.

Manufacturers have designed specific rollover protection structures in the form of cages to reduce the injury risk during crashes or rollovers. These cages, frames, or appendages are known to increase the safe space around the driver, to avoid severe injuries [2, 70]. Other rollover protection systems, such as quad-bars, tend to prevent the complete rollover that may trap the rider underneath the vehicle, leading to severe injury and/or asphyxiation. However, heavier rollover protective structures were found to decrease the vehicle's stability [2]. Zellner, in a simulation study, reported a risk/benefit proportion of 492% (95% CI 255%, 788%), bringing out a surprising conclusion that such rollover protection structures may decrease the vehicle's stability and increase the risk for rollover [65]. Furthermore, another study argues that these structures may hinder active dismount and increase the chance of being trapped under the vehicle in a rollover [70].

The rollover moment of a dynamic vehicle such as the quadbike depends on its center of gravity, which constantly shifts as the vehicle bounces and tilts on uneven surfaces. Therefore, the higher center of gravity, as seen in recreational quadbike designs, may be the reason to their predisposition to rollover when compared to utility quadbikes used in farming [48].

Another design feature that is believed to decrease the slope stability is the narrow vehicle track width. Edlund [2] showed that a 20-mm increase in the width contributed to preventing rollover even when the slope increased by a 32° angle. Designs with greater engine capacity predispose the vehicle to crashes [77]. Butts reported that riders on vehicles with engine displacement of more than 350 cc had more severe injuries than those riding on lower engine sizes [71].

#### Terrain-physical riding environment

##### Paved road

Despite being designed for off-road use with low-pressure tires and locked rear axles [1], QBs are not stable at high speeds on paved roads. Paved roads are asphalt- or concrete-covered roads that are inferior to rough terrain in their energy absorbing ability. It allows riding at higher speeds and consequently higher kinetic energy transfer upon impact. Riding on paved roads increased the risk for crashes fivefold compared to riders on uneven terrain, even after adjusting for riding frequency, gender, and multiple riders [58]. Riding on public roads and state highways had a higher risk of mortality than off-road use [49]. Paved road crashes predominantly result in collisions and ejections of riders, while those occurring on unpaved roads are more likely to result in rollovers [1, 55, 72]. Jennissen showed from a survey study how passengers increased the risk for crashes to threefold [58]. Unsafe riding behaviors were reported to cluster more often, with crash victims who reported to have been driving on paved roads and those who have taken in passengers [58]. Similarly, Campbell observed an increased risk of injury among riders riding at night [32].

##### Rural residence

Living on a farm was found to be significantly associated with a lower rate of helmet use and riding on paved roads, especially among farmers who used QBs for occupational purposes rather than recreational uses [58]. The availability of riding areas evidently induced more frequent QB usage and was more associated with higher injury rates [59]. Easy access to quadbikes when owned by the family was another factor that increased injury rates among rural children [44]. Familiarity with terrain was as crucial as riding experience in farmers' risk perception [53]. When compared to their urban counterparts, rural riders were more likely to use riskier terrains placed further away from emergency medical centers, which contributed to increased injury severity following crash events [59].

##### Organized riding parks and supervision

Off-highway vehicle parks are spaces solely dedicated to organized and controlled quadbike driving for recreational activities. These parks are known to enforce quadbike helmets and safety laws. Organized riding parks showed three times greater helmet use than public riding spaces [72]. Again, organized parks reported no injury victims below the age of 6 years, lower injury rates, and lesser severe head injuries than unorganized riding in Iowa, USA [72]. While organized parks make parental supervision mandatory for riders under the age 12, this is not the same in unorganized public riding spaces. Campbell reported higher crash rates among children riding without parental supervision [32].

##### Sociopolitical risk factors—legislation and enforcement

Similar to the measures available for controlling motor vehicle accidents, legislation and enforcement are also essential to reduce quadbike injuries. These measures include age restrictions on QB usage, mandatory helmet use, and banning the use of quadbikes on paved public roads. For example, age-restriction laws in Massachusetts State, USA, ensured that children under 13 years should not drive unsupervised and were restricted to vehicles with engine sizes less than 90 cc. The legislation brought a significant drop in hospitalization rates and emergency visits among children under 7 years of age [66, 73]. Such outcomes were not seen with other QB safety interventions, such as certification and licensing laws introduced in 2003 [12] or helmet laws, introduced in 2005 [42]. However, introducing legislations or laws alone might not make an impact unless they are followed with rigorous enforcement. Active enforcement showed a significant difference in mortality between violators and non-violators of state laws [10]. State laws’ violation due to ignorance among half of young respondents in Jennissen's study could support the observation [58].

## Discussion

The review synthesized evidence on risk factors related to QB crashes, with most evidence coming from the USA and focused on the pediatric population. This review shows how traditional intervention methods such as legislation and training alone had a weak influence on reducing QB injuries. Modifiable risk factors, such as increasing the age of driving initiation, reduced substance use, and use of organized riding parks, could reduce injuries. Riding practices, including avoiding passengers, avoiding nighttime riding, and using helmets, could reduce crashes and injuries among drivers and passengers. Vehicle modifications such as increased wheel base and limiting engine displacement could help reduce crash incidence.

### Strengths

#### Etiological factor vs risk factor

A systematic review of risk factors is beneficial for policymakers in understanding the etiology and the risk related to an outdoor sport such as QB riding. The findings will hopefully lay the basis to inform policymaking and to prioritize and justify safety interventions and resource allocation [74]. Stringent inclusion and exclusion criteria are pivotal to differentiate etiological factors and risk factors in systematic review studies. Etiological factors are those factors that are correlated with the outcome, without proven causality or temporality [17, 19, 74, 75]. Our review does not select studies that only report probable etiological factors. This weeds out cross-sectional studies that focus on injury prevalence. Such excluded studies were observational studies reporting risk prevalence without testing its association with the outcome. We included only those studies that identified risk factors and tested their association with outcomes. One must also note that evidence from observational studies may not prove causality but only hint at risk association with crashes and injuries.

#### Gray literature and engineering database

In addition to the medical databases used regularly for systematic reviews, such as PubMed, Scopus, and Embase, a wealth of information was also derived from the gray literature. Sources such as ProQuest Dissertations and Theses could provide high-quality evidence from theses and reports. Hopewell identified substantial evidence emerging from unpublished works in injury prevention reviews, thus reducing publication bias [76–78]. Databases from the engineering field, such as the IEEE Explore Digital Library, ASME Digital Collection or Transportation Research Information Services Database, also contributed to vehicle-related studies, which forms an essential part of the Man-Vehicle-Environment risk triad [17, 75]. Unfortunately, IEEE Explore has been exploited by very few systematic reviews for QB injury risk evidence [79].

#### Study data validity assessment for diverse study methods

The main weakness of reviewing different study designs in a narrative review is the low quality of evidence. Therefore, while trying to maintain the comprehensiveness of the evidence base, we also tried to ensure the quality of evidence from observational studies. Even though a single assessment tool could not assess the quality of a wide range of study methods, we applied the MMAT screening tool [29] for each study classification. This tool is widely used in systematic reviews involving mixed-method studies or heterogeneity of study methods [80–82]. The mixed-method assessment tool [29] for risk of bias assessment guaranteed high quality of evidence over a wide range of study designs. Thus, we have tried to develop a systematic review that identifies a comprehensive list of risk factors without compromising the quality of evidence.

While these assessment tools were adequate for population-based epidemiological studies, they could not access laboratory-based or experimental vehicle simulation tests [2, 65, 67, 69, 70]. Therefore, for such studies, a stringent inclusion criterion was applied to select only the studies that looked into prospective human injury as their outcome.

### Limitations

#### Absence of meta-analysis

Applying systematic review methodology to identify risk factors for vehicle injuries is limited by the nature of the studies searched. Analytical studies using retrospective registry data on injuries and incident reports dominated the search results in addition to primary data collected through surveys. Randomized trials were absent due to the post hoc nature of enquiry after injury. The trials were limited to intervention studies. The risk association observed in this review does not establish causality.

The heterogeneity of the study methods and the wider range of outcome measures prevented meta-analysis in this review. Many studies focused on deaths that resulted from QB crashes as the primary outcome [49, 59, 61, 83], while others reported injuries, either general injuries [32, 46] or specific injuries, such as traumatic brain injury [56, 63, 64], as the outcome. This variety of outcomes makes them unfit for meta-analysis. Moreover, this review included studies with different study methodologies, such as retrospective analytical studies [39, 41, 50, 56, 59–63, 66, 73], qualitative studies [53], cross-sectional observational studies [9, 32, 45, 46, 52, 58, 71], and laboratory-based simulation studies [2, 65, 67, 69, 70]. Thus, the diversity of outcomes and study methodologies ruled out meta-analysis or statistical treatment [27, 84].

#### Low sensitivity of systematic reviews in comprehensive risk identification—post-crash factors

The search terms developed for this systematic review attempted to identify the risk factors related to QB crashes. However, the review was not able to identify any significant post-crash risk factors. Post-crash risk factors increase the severity of injuries after the rider experiences a crash. This gap could be due to insufficient research using hospital data or due to system-related factors. We drew this conclusion when we did not obtain results from the lay search using the terms “postcrash” AND [outcome OR severity] AND [“QB” or “quad bike”]. This low sensitivity of our search strategy could be compounded by our exclusion of case studies and prospective case series, as they did not test the association between risk factors and outcomes. In addition, we excluded studies on post-crash factors at screening, as their outcomes were not crashes or injuries. Thus, post-crash factors would need another review with broader inclusion criteria.

### Applying risk association to prevention strategies

#### Regulation and standards

Legislation and enforcement are key public health measures to ensure the traffic safety environment (e.g., banning the production and use of certain designs) and the control of risky behaviors (e.g., driving under the influence of alcohol) to control injuries and deaths related to the use of quadbikes [85]. The review attempts to evaluate the impact of legislation and enforcement in the reduction in QB-related injuries. These legislations include laws restricting the use of certain quadbikes in terms of engine size, age, licensing, and paved roadway use restrictions. While shown to be effective in reducing injuries, mandatory helmet use and licensing laws were proved to be less effective [10]. In 1998, the USA put into force a comprehensive set of interventional programs aimed at the education and training of quadbike users called the QB Action Plan. It involved training incentives for QB owners, massive educational campaigns to enforce age restriction, and close supervision of QB sales to children and other awareness campaigns [11]. Sadly, this period also saw a sharp increase in fatal injuries due to QB crashes, especially in the pediatric population, attributed to the lapse of strict age restrictions and mandatory training at the point of sale [86, 87]. Interventions on education, training, and awareness without controlling vehicle use proved ineffective in curbing QB injuries [69, 88]. In the same vein, evidence shows that the introduction of regulations addressing personal and vehicle risk factors alone without accommodating environmental risk factors appeared to be inadequate in reducing injuries and deaths.

#### Training programs

Training programs to reduce the incidence of quadbike injuries are composed of operational knowledge on how to control the vehicle and how to mitigate the environmental risks and drivers’ hazards perceptions [89]. Risk perception and hazard perception training are known to increase compliance with safe driving behavior and the use of protective equipment [90]. Training could also sensitize riders to identify loss of control events that could lead to better reporting practice [9]. Studies exploring QB training outcomes noted that training alone did not reduce the risk for crash injuries and hospitalizations among quadbike users [44, 91, 92]. Although quadbike training and education programs targeting youth for safe QB driving, using school-based, experiential and game-based methods have been tested [44, 93, 94], their impact was not evaluated prospectively and temporality was not established. These studies might fail to assess and correctly estimate the reduction in low-severity crashes that do not reach the hospital or the adoption of safe driving practices [89, 95]. The training curriculum and content were either not elaborated [44, 91, 92] or were limited to basic driving skills and safety knowledge [93]. Some of the best practices adopted in motorcycle training and licensing, such as early age of training, compulsory training, graduated licensing, and long duration of training, have yet to be tested and implemented among QB riders [89, 96, 97]. In addition to basic riding skills and terrain awareness, courses could also focus on developing risk perception for safety behavior modification. The emphasis to make training a community-wide effort could also help in reaching and hopefully sustaining a positive shift in QB riding culture and riding behavior.

#### Indirect risk factors—helmet use

This review could not capture risk factors that indirectly influence riding behaviors or injury outcomes. While wearing protective equipment such as helmets has been proven to reduce head injury severity, some studies have explored the factors influencing helmet use. Qualitative studies showed a lack of perceived risk and helmet discomfort among users, which made a sizable portion of QB riders abandon their helmets [98, 99]. Riders who have undergone formal training on quadbike riding were found to be four times more likely to wear helmets than those who did not [92]. Similar observations on helmet use were reported among skiers [100] and recreational cyclists [101]. Bethea and Bohl observed how driving under the influence of alcohol made riders less likely to wear helmets [56, 60]. A similar influence of alcohol on helmet nonuse was found among motorbike and bicycle riders [102–105]. Jinnah came across a surprising finding that older girls were less likely to wear protective gear when compared to boys despite being the less affected gender in other age-groups [106]. Girls and women were found to be less willing to use helmets than bikers and two-wheelers [107–109].

#### Target groups and perspective

Research on QB injury is heavily skewed toward the pediatric population, with 32 percent of all studies targeted at children under 16 years. The focus on pediatric riders could be attributed to the fact that this age-group is at the highest risk of death and severe injury compared to other age-groups in the population [5, 24, 91]. Eighty percent of these studies targeted farm workers and rural populations, which reflects the occupational viewpoint of most of these studies.

#### Age of initiation

An immature age of driving initiation was found to predispose riders to more risky driving habits, including excessive speed and failure to wear protective gear [106, 110]. Risky driving behaviors, including excessive speeding, nonuse of helmets, and violating safety laws, have long been correlated with early driving initiation among car drivers [111, 112]. This correlation has been the basis for introducing a graduated driving licensing scheme among car and motorbike users [113, 114]. Unfortunately, similar regulations have not yet been introduced for recreational sports such as quadbike riding [97].

## Conclusion

This systematic review has successfully identified QB crash determinants and risk factors from observational studies. These determinants and risk factors were related to driver attributes, vehicle attributes, driving terrain, and sociopolitical factors. Despite the limitations known for observational studies, the findings provide adequate evidence to support policies and safety interventions aiming to reduce quadbike injuries. However, the review missed identifying post-crash system-level factors. Injury reduction intervention research should prioritize system-level risk factors, post-crash factors, and environmental factors. To address QB risk factors and reduce injuries, greater focus must be placed on different risk factors for QB crashes and injuries by involving multiple stakeholders. We must move beyond the “education, engineering and enforcement” approach and adopt the sustainable development goal approach.

## Data Availability

Data are available upon request.
